# Supplementary material for: Rapid Regulation of Human Mesenchymal Stem Cell Proliferation Using Inducible Caspase-9 Suicide Gene for Safe Cell-Based Therapy
Source: Int J Mol Sci. 2019 Nov 16;20(22):5759. doi: 10.3390/ijms20225759 (PMC6887989; doi:10.3390/ijms20225759)
Supplement: Supplementary file 1 [file ijms-20-05759-s001.pdf]

Supplementary Information

Rapid regulation of human mesenchymal stem cell proliferation using inducible caspase-9 suicide gene for safe cell-based therapy

Mari Tsujimura[1], Kosuke Kusamori[1,*], and Makiya Nishikawa[1]

[1] Laboratory of Biopharmaceutics, Faculty of Pharmaceutical Sciences, Tokyo University of Science, 2641 Yamazaki, Noda, Chiba 278-8510, Japan

Supplementary Figure S1.

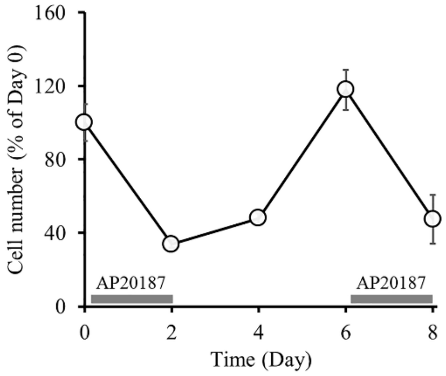

Figure S1. Regulation of the proliferation of UE7T-13/iC9 cells by AP20187. UE7T-13/iC9 cells were cultured in medium containing 0.05 nM AP20187 for day 0 to day 2 and day 6 to day 8 (Gray bars). Results are expressed as the mean ± SD of three samples.
